# A novel real-world ecotoxicological dataset of pelagic microbial community responses to wastewater

**DOI:** 10.1038/s41597-020-0496-5

**Published:** 2020-05-27

**Authors:** J. E. Ruprecht, W. C. Glamore, K. A. Dafforn, F. Wemheuer, S. L. Crane, J. van Dorst, E. L. Johnston, S. M. Mitrovic, I. L. Turner, B. C. Ferrari, S. C. Birrer

**Affiliations:** 1 Water Research Laboratory, School of Civil & Environmental Engineering, UNSW Sydney, Sydney, NSW 2052 Australia; 2 Evolution and Ecology Research Centre, School of Biological, Earth and Environmental Sciences, UNSW Sydney, Sydney, NSW 2052 Australia; 3 Department of Earth and Environmental Sciences, Macquarie University, Macquarie Park, NSW 2109 Australia; 4 Ferrari Lab, School of Biotechnology and Biomolecular Sciences, UNSW Sydney, Sydney, NSW 2052 Australia; 5 Freshwater and Estuarine Research Group, School of Life Sciences, University of Technology Sydney, Ultimo, 2007 NSW Australia

**Keywords:** Water microbiology, Civil engineering, Water resources, Environmental impact

## Abstract

Real-world observational datasets that record and quantify pressure-stressor-response linkages between effluent discharges and natural aquatic systems are rare. With global wastewater volumes increasing at unprecedented rates, it is urgent that the present dataset is available to provide the necessary information about microbial community structure and functioning. Field studies were performed at two time-points in the Austral summer. Single-species and microbial community whole effluent toxicity (WET) testing was performed at a complete range of effluent concentrations and two salinities, with accompanying environmental data to provide new insights into nutrient and organic matter cycling, and to identify ecotoxicological tipping points. The two salinity regimes were chosen to investigate future scenarios based on a predicted salinity increase at the study site, typical of coastal regions with rising sea levels globally. Flow cytometry, amplicon sequencing of 16S and 18S rRNA genes and micro-fluidic quantitative polymerase-chain reactions (MFQPCR) were used to determine chlorophyll-a and total bacterial cell numbers and size, as well as taxonomic and functional diversity of pelagic microbial communities. This strong pilot dataset could be replicated in other regions globally and would be of high value to scientists and engineers to support the next advances in microbial ecotoxicology, environmental biomonitoring and estuarine water quality modelling.

## Abstract (summary)

**Table:** 

Measurement(s)	water composition • total dissolved solids • conductivity of water • pH • concentration of oxygen in water • Total Organic Carbon • dinitrogen • phosphorus atom • ammonia • nitrate • nitrite • biological oxygen demand • chlorophyll a • Cell Density • Algae • Bacteria • hydrogen sulfide • Toxicity • rRNA_16S • rRNA_18S • abundance of nutrient cycling genes • abundance of antibiotic resistance genes
Technology Type(s)	water quality unit • water testing suite • dilution method • autofluorescence • flow cytometry method • whole effluent toxicity testing • DNA sequencing • microfluidic quantitative polymerase chain reaction
Factor Type(s)	effluent concentration • salinity levels
Sample Characteristic - Organism	Bacteria • algae
Sample Characteristic - Environment	waste water • estuary • sea coast • saline water • fresh water body
Sample Characteristic - Location	Hunter River

Machine-accessible metadata file describing the reported data: 10.6084/m9.figshare.12221738

## Background & Summary

The world is facing a global water quality crisis^1,2^. The vast majority (more than 80%) of global wastewater is released directly into natural waterways, resulting in widespread pollution^3^. The most frequent contaminants are domestic waste (~2 million tonnes per day), industrial wastes and chemicals, agricultural pesticides and fertilizers^2,4^. The implications of wastewater discharges include, but are not limited to: degraded aquatic ecosystems^5,6^; decreased biodiversity^7^; increased greenhouse gas emissions^8,9^; and a wide range of detrimental impacts to human health^10^. Global wastewater volumes are increasing at unprecedented rates as a result of population growth, rapid urbanisation and economic development, and these drivers are concentrated in coastal regions^11–14^. This worldwide trend poses immediate management challenges if we are to prevent further damage to sensitive aquatic ecosystems, human health and water security^15^.

Globally, comprehensive datasets that characterise the impacts of wastewater discharges to water quality in natural aquatic environments are generally lacking^2^. Based on a comprehensive review of data published in 181 countries^11^, the authors found that only 55 countries had any data available on wastewater generation, treatment and use, and much of this information was dated (i.e., pre-2008). Significant data gaps exist on the linkages between the physical, chemical and biological characteristics of many urban surface water environments that receive wastewater discharges^16–18^. In highly developed countries where the largest percentage of treated domestic wastewater is currently discharged directly to natural waterways – for example: Australia (85%; 1,234 treatment plants), North America (75%; 14,748 treatment plants) and Europe (71%; >18,000 treatment plants) – an understanding of the impacts of effluent discharges on ecosystem health is still in its relative infancy^11^. These pressure-stressor-response relationships are particularly difficult to disentangle in estuaries, due to highly variable physio-chemical conditions^19^. To effectively address increasing concerns regarding wastewater discharge to natural aquatic systems worldwide, comprehensive data is urgently needed from real-world observations. These data can be used to investigate and understand the pressure-stressor-response linkages between treated effluent discharges and natural aquatic environments.

Pelagic microbial communities are extremely sensitive to rapid changes in their environment making them ideal indicators of water quality processes and functioning^19–21^. They are also ubiquitous in natural aquatic environments and play an important role in nutrient and organic matter cycling^22^. Traditional microbial ecotoxicological studies have relied on the combination of single algal species toxicity testing and chemical surveys to ascertain the aggregate toxic effect of whole effluent wastewater discharge on microalgae, rather than attempting to quantify both diversity and function of entire microbial communities at the same time^21^. The combination of community-level testing and environmental ‘omics moves beyond the scenario of single species toxicity testing and provides the opportunity to determine real-world community interactions and shifts in response to wastewater.

In this study we have incorporated recent advances in water quality science and assessment techniques to characterise the ecotoxicological response of tertiary-level treated effluent following discharge in temperate estuarine environments. The integrated field and laboratory assessments were completed on the Hunter River estuary located on the New South Wales (NSW) coastline in southeast Australia. The dataset obtained is specifically targeted at the dynamics and health of pelagic microbial communities at a practical scale, with these novel observations having the potential to provide new insights and understanding of nutrient and organic matter cycling. Whole Effluent Toxicity (WET) testing was performed to highlight the effects of mixing treated effluent within freshwater and saltwater environments and to identify potential tipping points that both inhibit and stimulate the growth of microalgae and microbial communities. Two salinity regimes were chosen to include future scenarios based on a predicted increasing salinity of the Hunter River with rising sea levels. All water samples were subjected to sequencing for 16S and 18S rRNA genes to measure changes in microbial community structure. Flow cytometry was used to enumerate chlorophyll-a and total bacterial cells and to estimate their size. The abundance of genes associated with nutrient cycling, antibiotic resistance and the identification of pathogens that would be harmful to human health were determined using microfluidic quantitative polymerase-chain reactions (MFQPCR) at the microbial community-level.

## Methods

### Study area

**Fig. 1:**
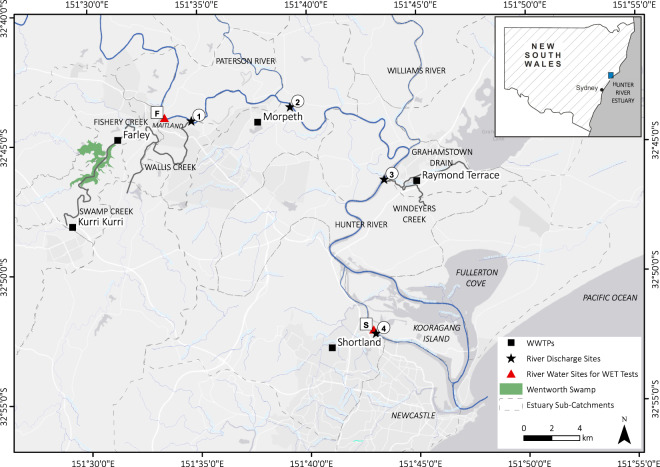
Hunter River estuary study area and WWTP river discharge sites for Kurri Kurri, Farley, Morpeth, Raymond Terrace and Shortland shown as circled numbers. River water collection sites used for WET test dilutions are shown as boxed letters.

The Hunter River estuary (151.8°E, 32.9°S) is situated on the temperate southeast coastline of NSW, Australia (Fig. 1). The Hunter River estuary is a typical wave-dominated, mature barrier estuary^23^ with a large tidal pool that extends 60 km inland to its tidal limits. The Hunter River estuary has semi-diurnal tides and a mean tidal range of approximately 1.2 m. Catchment inflows to the estuary via the Hunter River and its two main tributaries – the Paterson and Williams Rivers – are regulated by dams and weirs.

The Hunter River catchment covers an area over 22,000 km^2^ and is typical of many other developed coastal regions globally in that it has been extensively modified by human activity and multiple land uses^24^. The upper catchment is predominately agricultural land, whereas the lower catchment around the Port of Newcastle includes extensive urban and industrial areas, entrance dredging and training, the world’s largest coal export terminal and a growing multi-purpose cargo hub. Despite these pressures, the Hunter River estuary still supports significant areas of estuarine habitat such as mangroves, saltmarsh, inter-tidal and sub-tidal soft sediment shoals, as well as a Ramsar listed wetland of international significance^25^.

The Hunter River estuary receives diffuse water pollution and nutrients from the catchment, as well as high nutrient point loads from several major wastewater treatment plants (WWTP) capable of servicing a population of approximately 200,000 people across the floodplain. Generally, these WWTP provide tertiary-level wastewater treatment designed to remove excess nutrients of nitrogen and phosphorus. Unallocated treated effluent is discharged either directly, or indirectly via tributary channels, to the tidal zone of the Hunter River. The tributary channels in this study are freshwater creek systems as they are excluded from tidal flows via one-way floodgates and form part of the expansive Lower Hunter Flood Mitigation Scheme.

The study sampling points were located in the upper, mid and lower portions of the Hunter River estuary (Fig. 1). In the upper Hunter River estuary, the Swamp-Fishery-Wallis Creek system near Maitland, receives unallocated treated effluent from the townships of Kurri Kurri (3.4 ML/day) and Farley (5.6 ML/day). Kurri Kurri WWTP discharges into Swamp Creek which flows into Wentworth Swamp, a large, low-lying permanent waterbody, which in turn discharges into Fishery Creek. Farley WWTP discharges into Fishery Creek downstream of its confluence with Wentworth Swamp, and this flows into Wallis Creek which discharges into the Hunter River estuary (Fig. 1, river discharge site 1). Unallocated treated effluent from Morpeth WWTP (10 ML/day) is discharged directly into the Hunter River, approximately 3 km downstream of Wallis Creek (Fig. 1, river discharge site 2). Unallocated treated effluent from the Raymond Terrace WWTP (7.3 ML/day) is discharged to the Hunter River via Grahamstown Drain and Windeyers Creek (Fig. 1, river discharge site 3). The Shortland WWTP (9.6 ML/day) discharges unallocated treated effluent directly to the Hunter River South Arm (Fig. 1, river discharge site 4). A description of site characteristics for each WWTP river discharge site (Fig. 1) is provided in Table 1.

**Table 1:** Description of WWTP river discharge sites.

Discharge site	Distance from ocean entrance (km)	Estimated residence time (days)^*^	Salinity range (ppt)^**^	50^th^ percentile salinity (ppt)
1	55	5	0.3–0.4	0.3
2	45	8	0.2–1.8	0.3
3	29	6	0.1–17.2	1.5
4	11	6	0.4–33.4	26.0

^*^Based on 50^th^ percentile modelled flows and dispersion coefficients^24^. ^**^5^th^ and 95^th^ percentiles calculated from a 110-year salinity timeseries simulated using a calibrated and validated hydrodynamic and salinity model of the Hunter River estuary.

### Microbial field surveys

Water samples were taken on two (2) separate occasions in the Austral summer (November 2016 and February 2017) from five (5) WWTP outfalls along the Hunter River estuary, including Kurri Kurri, Farley, Morpeth, Raymond Terrace and Shortland. Water samples were collected upstream and downstream of each outfall at different distances totalling 20 sampling points (see Table 2). Note that water at Shortland was only sampled from the river immediately upstream of the Shortland WWTP outfall – downstream sampling was not logistically possible and not relevant as the WWTP was not discharging at the time of the investigation. All 20 sites were sampled within the same two-day time window on both sampling occasions with three (3) replicates collected per site for sequencing of microbial (prokaryotic and eukaryotic) communities and a single replicate for water quality measurements.

**Table 2:** Microbial field survey site details, including sample IDs, site names, the main WWTP contributing to the water collected and whether the site was in a creek or the main channel of the Hunter River.

WWTP	Site name	Sample ID	Type	Distance from WWTP
	Kurri Kurri	KK-UP (BG)	Creek	50 m upstream of outfall (background)
Kurri Kurri	Kurri Kurri	KK-DN	Creek	50 m downstream of outfall
	Farley	FAR-UP (BG)	Creek	50 m upstream of outfall (background)
Farley	Farley	FAR-DN	Creek	50 m downstream of outfall
Kurri Kurri + Farley	Wallis Creek	WLC (WLC-DN)	Creek	1500 m upstream of river discharge site 1
	Wallis Creek	WLC-UP (BG)	River	50 m upstream of river discharge site 1 (background)
Kurri Kurri + Farley	Wallis Creek	WLC-0m	River	River discharge site 1
Kurri Kurri + Farley	Wallis Creek	WLC-50m	River	50 m downstream of river discharge site 1
Kurri Kurri + Farley	Wallis Creek	WLC-500m	River	500 m downstream of river discharge site 1
	Morpeth	MOR-UP (BG)	River	50 m upstream of river discharge site 2 (background)
Morpeth	Morpeth	MOR-0m	River	River discharge site 2
Morpeth	Morpeth	MOR-50m	River	50 m downstream of river discharge site 2
Morpeth	Morpeth	MOR-500m	River	500 m downstream of river discharge site 2
	Windeyers Creek	WC-UP (BG)	Creek	50 m upstream of outfall (background)
Raymond Terrace	Windeyers Creek	WC-DN	Creek	50 m downstream of outfall
	Raymond Terrace	RT-UP (BG)	River	50 m upstream of river discharge site 3 (background)
Raymond Terrace	Raymond Terrace	RT-0m	River	River discharge site 3
Raymond Terrace	Raymond Terrace	RT-50m	River	50 m downstream of river discharge site 3
Raymond Terrace	Raymond Terrace	RT-500m	River	500 m downstream of river discharge site 3
Shortland	Shortland	SL-0m	River	River discharge site 4

Sites are listed from highest to lowest chainage from the ocean entrance, with increasing salinity in the river. Note that the WWTP has been abbreviated in the data records as follows: Farley = FAR, Kurri Kurri = KK, Morpeth = MOR, Raymond Terrace = RT, Shortland = SL. Additional abbreviations include: Wallis Creek = WLC, Windeyers Creek = WC, Background = BG, Downstream = DN.

Specifically, surface water was collected in 2 L sterile Whirl-Paks^®^ stored on ice in the dark until filtering within 24 hours. To capture all microbial cells and fragments in the water samples for DNA extraction and sequencing, samples were homogenised by repeated inversion and 500 mL of water was filtered through a 0.22 µm Express PLUS Polyethersulfone membrane (Millipore) using a hydraulic pump. Filter units were sterilised before use and rinsed with ethanol between water samples. In some cases, when the water samples contained excess organic material, the filters were clogged before 500 mL could be filtered, and the volume that had been filtered was noted for later adjustment of the data. Filters were rolled, inserted into bead tubes from the DNeasy PowerWater Kit (Qiagen) and frozen at −80 °C until DNA extraction and sequencing.

For water quality measurements, water samples were simultaneously obtained using standard bottles provided by a NATA accredited facility for analysis of total suspended solids (TSS), total organic carbon (TOC), total nitrogen (TN), total phosphorous (TP), ammonia (NH_3_), nitrate and nitrite (NO_x_), biological oxygen demand (BOD), chlorophyll-a, and hydrogen sulphide (H_2_S). Water samples were stored on ice in the field and then sent for testing on the same day they were sampled. Additional environmental data, including pH, dissolved oxygen (DO), and electrical conductivity (EC) were measured using a calibrated water quality unit (Horiba) at each site. The water quality unit was calibrated before and after each sampling trip.

### Whole Effluent Toxicity (WET) testing

Whole Effluent Toxicity (WET) testing was conducted within the laboratory facilities of the Sydney Institute of Marine Science (SIMS) in Chowder Bay, Sydney, Australia. WET tests were completed for algal single-species and whole microbial communities in a fully-crossed experimental design created using UV-disinfected effluent from the five (5) WWTP sampled. Following standard protocols for WET testing as published by the US EPA and the ANZECC/ARMCANZ water quality guidelines (2000), five effluent concentrations were selected along with a control. The concentrations comprising 0% effluent (control), 0.1% effluent, 1% effluent, 10% effluent, 50% effluent, 90% effluent and 100% effluent.

Sample collection, preparation and testing was completed over three (3) weeks in May 2017. Disinfected effluent from each WWTP and river water samples were collected on the first day of each test week and stored in a dark constant temperature (25 °C) room O/N to allow for water temperature adjustment to the testing conditions. On each sampling occasion, a total of 30 L of disinfected effluent from each WWTP and 60 L from each river site was collected to create the dilutions for the WET tests.

#### Single species

The single-species WET tests were completed based on standard procedures – US EPA Test Method 1003.0^26^ and the Environment Canada test method^27^ – using a 4 to 7-day old culture of the freshwater unicellular green algae *Raphidocelis subcapitata* (formerly also known as *Selenastrum capricornutum* and *Pseudokirchneriella subcapitata*) obtained from the NSW Department of Planning, Industry and Environment (formerly NSW Office of Environment and Heritage). Prior to WET testing, cells of *R. subcapitata* were washed three (3) times with artificial soft-water to remove culture media and extracellular substances. For this step, the algal culture was centrifuged at 700 g for seven (7) minutes, the supernatant discarded, and the pellet resuspended in artificial soft-water. Algal cells were counted using a haemocytometer and each test sample was inoculated with approximately 3 × 10^4^ microalgal cells/mL.

For the single-species WET tests, 200 mL dilutions were prepared using UV-disinfected effluent (filtered at 0.45 µm) from all five (5) WWTP and artificial soft-water (filtered at 0.22 µm). Artificial soft-water used for the tests was created by the addition of sodium bicarbonate (*NaHCO*_3_), calcium sulfate dihydrate (*CaSO*_4_·2*H*_2_*O*), magnesium sulfate heptahydrate (*MgSO*_4_·7*H*_2_*0*), and potassium chloride (*KCl*) to milli-Q water. The artificial soft-water was adjusted to the hardness of each effluent water type on the starting day of the WET tests. Total hardness as CaCO_3_ for effluents from each WWTP included: Kurri Kurri (106 mg/L); Farley (257 mg/L); Morpeth (78 mg/L); Raymond Terrace (65 mg/L); and Shortland (168 mg/L). Three (3) replicates of 50 mL each were added to 100 mL conical flasks and one (1) replicate of 50 mL was added to a 100 mL glass beaker. The flasks and beakers were covered with a 0.4 mm mesh to avoid any airborne particles contaminating the samples. Further, 200 µL of 2.1 g/L sodium nitrate (1.5 mg $N{O}_{3}^{-}$/L) and 200 µL of 0.22 g/L potassium dihydrogen phosphate (0.15 mg $P{O}_{4}^{3-}$/L) was added to each sample to provide sufficient nutrients for exponential algal growth.

The test samples were incubated for 72 hours at continuous daylight conditions. During the tests, full-spectrum daylight fluorescent lighting (36 W) provided a light intensity of 71.6 W/m^2^. The shelves used for the tests were lined with aluminium foil and samples were randomised once a day to maximise light exposure. At the time of inoculation (time point 0 h), the concentrations of NH_3_, NO_x_, TKN, TN, and TP, as well as water hardness were measured in each effluent type using standard inorganics and nutrients water testing suites. At the end of every nominal 24-hour period, test samples were mixed by swirling (conical flasks) or with pipette tips (beakers). 100 µL from each conical flask was taken to determine cell counts using flow cytometry, and water quality microsensors (Unisense) were used to measure pH and DO levels in the beakers.

#### Whole microbial community

For the community-level microbial WET tests, dilutions were prepared using unfiltered UV-disinfected effluent from all five (5) WWTP and ulfiltered river water from two (2) locations in the Hunter River estuary, including a freshwater site (salinity < 1 PSU, river site ‘F’ in Fig. 1) and a saltwater site (salinity > 30 PSU, river site ‘S’ in Fig. 1). These two (2) salinity regimes were chosen to include future scenarios based on a predicted increasing salinity of the Hunter River with rising sea levels. The community-level WET tests were run in triplicate 2 L plastic beakers for 72 hours at a 12/12 hour day/night light cycle. During the tests, LED-600 aquarium lights (BeamsWork) provided conditions suitable for the growth of photoautotrophic microbes, having a light frequency of 10,000 K (white LED) and 460 nm (blue LED), equivalent to 1,340 lumens and a light intensity of 65.1 W/m^2^ (assuming a luminous efficacy of 40.98 lm/W). As for the single-species tests, the shelves used for the tests were lined with aluminium foil and the beaker positions on the shelves were randomised once a day to maximise the light exposure in each beaker.

At the time of inoculation (time point 0 h), concentrations of NH_3_, NO_x_, TKN, TN, and TP were measured in each effluent type and river site sample. At the end of every nominal 24-hour period, beakers were mixed with a glass stirring rod and samples from each beaker were taken for further analysis, including 1 mL of water for microbial cell counts using flow cytometry, and approximately 500 mL of water, collected in sterile Whirl-Paks and stored on ice in the dark until filtering, for DNA extraction and sequencing. Note that filtering for DNA extraction during the WET tests followed the same protocol previously described for the microbial field surveys. Further, water quality microsensors (Unisense) were used to measure pH, DO and H_2_S at the sampling times (i.e. every 24 hours) for each effluent type and dilution, except Shortland WWTP due to technical difficulties. Note that DO at time point 0 h was only measured for Raymond Terrace and Morpeth effluent types due to a temporary fault in the DO sensor.

#### Biological oxygen demand

At the end of the community-level WET tests (time point 72 h), water was sampled from two (2) replicates of each effluent type and dilution for nutrient (NH_3_, NO_x_, TKN, TN, TP) analysis, and 200 mL of the remaining water was used to estimate the BOD of the microbial communities. The water sampled for the BOD tests were capped in standard BOD bottles and incubated for 2.5 hours in the dark. DO measurements were taken at the start of the BOD tests (T0) and after 2.5 hours incubation in the dark (T1).

### Flow cytometry

#### Staining optimization

The nucleic acid dye SYTO9 (ThermoFisher Scientific), which has been shown to preferentially stain live and dead total bacteria cells, was tested at varying dilutions (1:80, 1:500, 1:1000 *v/v* of the dye’s stock solution and dimethyl sulfoxide (DMSO))^28,29^. All dilutions were tested using triplicate positive (stained) and negative controls (no stain). After stain optimisation using an analogue river water sample, all aliquots were subsequently stained using 0.2 µL of SYTO9 (1:1000 *v/v* dilution of commercial stock solution with DMSO) and incubated for 15 minutes in the dark at room temperature prior to analysis^28^. Note the differentiation and quantification of both live and dead cells, using Propidium Iodide, was purposely omitted, since sequencing typically incorporates both live and dead cells, and the results were aimed at determining the total number of bacteria cells (live and dead) present in the sample.

#### Flow cytometry analysis

All cell count samples were analysed using a LSRFortessa SORP (BD Biosciences) flow cytometer with the FACSDiva software (v8.0.1) in the Mark Wainwright Analytical Centre (Flow Cytometry Facility) at UNSW Sydney. Cell count samples from the single-species and microbial community WET tests were stored on ice until flow cytometry analysis. The samples analysed in May 2017 (‘Fresh’) were left unstained and the autofluorescence from chlorophyll-a present in the live algal cells was used to determine cell densities. The chlorophyll-a fluorescence assays were designed to compare the growth response of single-celled algae between the two (2) WET setups. Additional water samples from the WET tests were stored at −20 °C and later used to quantify the total bacterial cell densities in February and March 2019 (‘Frozen’). This was done to distinguish between the bacterial and algal (chlorophyll-a) cell populations in the water samples.

All cell count samples were separated into 200 μL aliquots across eight (8) microplates (96-wells) by effluent type (Kurri Kurri and Farley, Morpeth and Raymond Terrace) and sampling time (0 h, 24 h, 48 h, 72 h). The Frozen samples were thawed quickly (at 37 °C) to avoid cell loss during the thawing process^30^, and after thawing, were mixed via manual shaking for 10 s. The defrosted 200 μL aliquots in the microplate wells were stained and analysed automatically in standard-throughput mode.

A threshold of 200 cell counts was set on the side-scattered (SSC) light detector to exclude noise from non-algal and unstained bacterial cell particles. All readings were collected as logarithmic signals at a flow rate of 2.0 µL/s. Maximum events were set to 10,000,000 to ensure the counting of all cells within a 60 µL sample (100 µL for the Fresh samples). Before cell counting, all samples were mixed twice-through by mechanical pipetting up and down of 100 µL of sample at a mixing rate of 180 µL/s. Note there was no cell count data available for the community-level WET tests containing the Shortland effluent type due to complications with the flow cytometer at the time of the analysis.

#### Enumeration and size

**Fig. 2:**
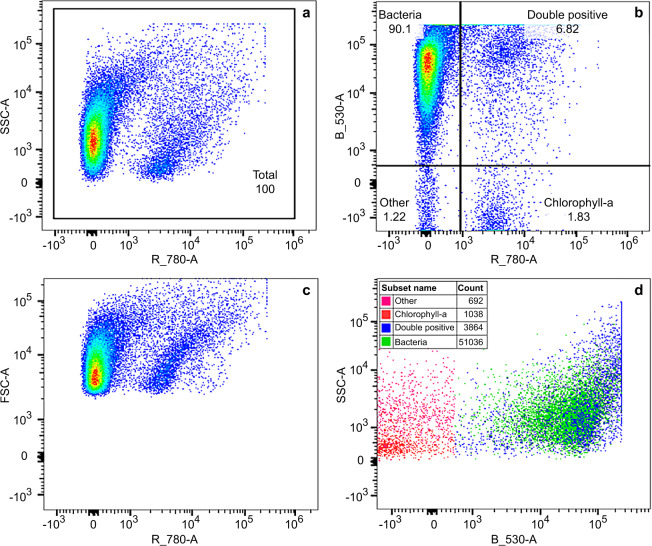
Example cytograms of bacterial and chlorophyll-a populations resolved by fluorescence for 50% dilution of Kurri Kurri effluent type in freshwater (replicate 1) at 48 hours, February and March 2019 dataset. A bivariate density plot of SSC vs red fluorescence (chlorophyll) shows the total population (**a**). Regions on the green vs red fluorescence plot were defined for bacteria (Bacteria), cells containing chlorophyll and SYTO9 stain (Double positive), cells containing only chlorophyll (Chlorophyll-a) and all other cells (Other) (**b**). Double positives were counted as total bacteria due to the separation of cells shown in SSC vs green fluorescence (**d**).

The 488 nm laser was used for excitation of both SYTO9 stain and chlorophyll-a fluorescence. The detection was measured using emission filters for green (530 nm ± 15 nm) and red (780 nm ± 60 nm) fluorescence. Determination of the region within the SSC vs chlorophyll bivariate density plot, which included live algal cells containing chlorophyll-a, was done prior to the WET tests, using algal cell cultures and negative controls. Further, determination of the region defined for total bacteria within the SSC vs green fluorescence plot, was done during staining optimisation, using positive (stained) and negative (unstained) controls. Example cytograms of bacterial and chlorophyll-a populations resolved by fluorescence for 50% dilution of Kurri Kurri effluent type in freshwater (replicate 1) at 48 hours are provided in Fig. 2. Figure 2a shows the total population which is separated into quadrants within the green vs red fluorescence plot (Fig. 2b). The region defined for total bacteria is labelled “Bacteria”, while the region for cells containing chlorophyll and SYTO9 stain is labelled “Double positive”. The double positives were counted as bacteria due to the separation shown in SSC vs green florescence (SYTO9 stain) (Fig. 2d). Final cell counts were determined as the number of events within the previously selected regions. Presentation of the data as bivariate density plots (Fig. 2) enabled the best distinction between stained bacteria and chlorophyll-a. Following enumeration of the microbial cells, size was estimated using CountBright absolute counting beads (200 nm, 500 nm, 800 nm, 1 um, 3 um, 6 um) as volumetric standards. There may be some variation in the size estimates caused by the way cells are placed during the analysis.

### DNA extraction and sequencing

DNA was extracted using the DNeasy PowerWater Kit (Qiagen) following manufacturer protocols and stored at −80 °C until sequencing. Bacterial communities were identified using the 27f (5′-3′: AGAGTTTGATCMTGGCTCAG^31^) and 519r (5′-3′: GWATTACCGCGGCKGCTG^32^) primers for the V1-V3 region of the 16 S rRNA gene and Illumina™ MiSeq. 2 × 300 base pair (bp), paired-end v2 sequencing runs across two (2) lanes. Eukaryotic composition was determined using the 1391 f (5′-3′: GTACACACCGCCCGTC) and EukBr (5′-3′: TGATCCTTCTGCAGGTTCACCTAC) primer set^33^ for the V9 region of the 18S rRNA gene and Illumina™ MiSeq. 2 × 150 bp paired-end v3 sequencing runs across two (2) lanes. All sequencing was done at the Ramaciotti Centre for Genomics (UNSW Sydney). All raw sequence data are publicly available through the National Center for Biotechnology Information (NCBI)^34^ under SRA study accession SRP224901. The SRA data record includes 1,596 experiments derived from 1,476 samples.

### Microfluidic quantitative polymerase chain reaction (MFQPCR)

The absolute abundance of nutrient (carbon, nitrogen, phosphorus and sulfur) cycling genes and genes associated with pathogens and antibiotic resistance were determined using microfluidic quantitative polymerase chain reaction (MFQPCR) on the Fluidigm platform. Like traditional qPCR, this method enables the determination of gene abundances through the measurement of fluorescence after each PCR cycle, but eliminates much of the manual pipetting from the sensitive qPCR reaction. Its use in ecological research in various environments has significantly increased in recent years, with a focus on fast and reliable detection of pathogens^35–37^.

Gene abundances were measured in two (2) out of three (3) replicates collected for microbial analyses from both the field surveys and WET tests. Suitable primers, as defined by^38^, were selected from the literature. Five (5) different gBlock gene fragments (Integrated DNA Technologies^39^) were designed as standards for the assays using targeted sequences for each primer pair sourced from NCBI (refer to Table 3) using Primer-BLAST^40^. Standard curves were generated using a dilution series of 10^1^ to 10^7^ copies/µL. Exact DNA concentrations of the samples were determined spectrophotometrically using the PicoGreen double-strand DNA kit (Life Technologies) on the ClarioSTAR^®^ microplate reader (BMG Labtech) and samples were diluted to a final DNA concentration of approximately 7–8 ng/µL. To alleviate sample-specific inhibition^41^, DNA extracts were mixed with T4 gene 32 protein (New England Biolabs^®^), to a final concentration of 80 ng/uL. Specific target amplification (14 cycles) and MFQPCR were conducted at the UNSW Sydney Ramaciotti Centre for Genomics, as per 38 using Evagreen^®^ chemistry and the 96.96 Fluidigm Dynamic Array™ Integrated Fluidic Circuit. Thermocycling consisted of 95 °C for 1 min, followed by 35 cycles of 96 °C for 5 s and 60 °C for 20 s or 25 s, followed by melt curve analysis for 60–95 °C at a ramp rate of 1 °C/3 s. Reactions were conducted in triplicate in 6.7 nL volumes with a final primer concentration of 700 nM.

**Table 3:** MFQPCR primers targeting nutrient cycling, pathogens and antibiotic resistance genes.

Primer pair	Category	Forward primer sequence (5′-3′)	Reverse primer sequence (5′-3′)	Reference	Standard	Length (bp)
16S	bacteria	GTGSTGCAYGGYTGTCGTCA	ACGTCRTCCMCACCTTCCTC	^48^	AM910662.1	145
18S	eukaryotes	AAGGAAGGCAGCAGGCG	CACCAGACTTGCCCTCYAAT	^49^	KF297603.1	150
Acc_ppk1	phosphorous pathway	GATGACCCAGTTCCTGCTCG	CGGCACGAACTTCAGATCG	^50,51^	KP738079.1	133
ampC	antibiotic resistance	TGAGTTAGGTTCGGTCAGCA	AGTATTTTGTTGCGGGATCG	^52^	CP002729.1	98
AOA_8	nitrogen pathway	CTATTCATAGTTGTAGTTGCTGTAA	ATGTAGTCTCCTGCGTTGAT	^38^	KX181620.1	80
AOB_1	nitrogen pathway	GTCTCCATGCTCATGTTC	GGAAAGCCTTCTTCGCC	^38^	KX683107.1	134
ciaB	pathogen	GCGTTTTGTGAAAAAGATGAAGATAG	GGTGATTTTACTTTCATCCAAGC	^53^	HE978252.1	80
dsrA	sulphur pathway	ACSCACTGGAAGCACG	GGTGGAGCCGTGCATGTT	^54^	KM241895.1	222
ftsZ	pathogen	CTGGTGACCAATAAGCAGGTT	CATCCCATGCTGCTGGTAG	^53^	CP018103.1	60
mecA	antibiotic resistance	CTGATGGTATGCAACAAGTCG	TGAGTTCTGCAGTACCGGATT	^52^	CP000255.1	96
napA_14	nitrogen pathway	ATGTGGGTGGAGAAGGA	TGAAGCGCTTGGAGAATT	^38^	LN901633.1	130
napA_3	nitrogen pathway	CCCAATGCTCGCCACTG	CATGTTKGAGCCCCACAG	^55^	CP014774.1	129
narG_1	nitrogen pathway	GACTTCCGCATGTCRAC	TTYTCGTACCAGGTGGC	^55^	DQ481141.1	68
narG	nitrogen pathway	TAYGTSGGGCAGGARAAACTG	CGTAGAAGAAGCTGGTGCTGTT	^56^	HM104383.1	110
nifD_33	nitrogen pathway	TGCCGTTCCGCCAGATGCA	AGATGGCGAAGCCGTCATAGC	^38^	CP010523.2	69
nifH_32	nitrogen pathway	GGCGTCATCACCTCGATCA	GCATAGAGCGCCATCATCTC	^38^	AP017605.1	176
nirK	nitrogen pathway	ATYGGCGGVCAYGGCGA	GCCTCGATCAGRTTRTGGTT	^57^	KX036332.1	164
nirS_1–3	nitrogen pathway	CCTAYTGGCCGCCRCART	GCCGCCGTCRTGVAGGAA	^58^	AB164133.1	257
nirS_ef	nitrogen pathway	CACCCGGAGTTCATCKTC	ACCTTGTTGGACTGGTGGG	^55^	JN257972.1	173
norB_79	nitrogen pathway	GAATACTGGCGTTGGT	ATACTTCAAAGAAGCCTTC	^38^	CP012027.1	55
nosZ_2	nitrogen pathway	CGCRACGGCAASAAGGTSMSSGT	CAKRTGCAKSGCRTGGCAGAA	^59^	KJ137778.1	269
nrfA_2	nitrogen pathway	CACGACAGCAAGACTGCCG	CCGGCACTTTCGAGCCC	^55^	AM408255.1	68
phoD	phosphorous pathway	GCCATTTATGCCGACACCT	TCCGATAGGCAGGCACATT	^60^	XM_002179950.1	130
phoNC	phosphorous pathway	CGGCTCCTATCCGTCCGG	CAACATCGCTTTGCCAGTG	^61^	AE008922.1	155
phsA	sulphur pathway	CGACCAGGACCTCATGCC	CTACCTGACCCCTGCTTTGC	^62^	CP002297.1	106
plc	pathogen	CATCAGTTGGAAAGAATGTAAAAGAAC	TGATTCCAAAATACATGTAGTCATCTG	^53^	DQ184163.1	96
rubisco_bact_cbbL	carbon pathway	AAGGAYGACGAGAACATC	TGCAGSATCATGTCRTT	^63,64^	*omitted*	—
rubisco_diatom	carbon pathway	GATGAYGARAACATCAACTC	TAAGAACCCTTAACYTCACC	^65^	EF143298.1	113
rubisco_haptophyte	carbon pathway	GAGAGCGTTTCCTATTCTC	CACGTGCGTACATTTCTTC	^65^	KF536380.1	117
rubisco_synecho	carbon pathway	CATCAAGCTGTCCGAG	TGTTGGCYGTGAAGCC	^65^	JN692350.1	161
sir	sulphur pathway	CCGTGTACTCCTCAACAAGATG	CCAATTCTGCCATGTAAGGAC	^66^	XM_002289791.1	101
stx2	pathogen	TCTGGCGTTAATGGAGTTYAG	GTGACAGTGACAAAACGCAGA	^53^	AB046175.1	78

## Data Records

The data format and metadata for the full archived datasets^34,42^ is provided in Tables 4–6. The design table in each dataset provides the lookup reference for each sample analysed for raw 16S and 18S rRNA gene sequences uploaded to the NCBI SRA database.

**Table 4:** Dataset record – Field Microbial Survey.

Data Type	Data File Tab	File Format
Design Table	Mapping	Col 1/2 (A/B)**–**Unique sample ID/name Col 3 (C)**–**Dataset Col 4 (D)**–**Date Col 5/6 (E/F)**–**Location/ID Col 7/8 (G/H)**–**Latitude/Longitude Col 9 (I)**–**WWTP Col 10 (J)**–**Distance from WWTP Col 11/12 (K/L)**–**Outfall location/ID Col 13 (M)**–**Interaction Col 14/15 (N/O)**–**Type/ID Col 16 (P)**–**Time point ID Col 17 (Q)**–**Replicate Col 18 (R)**–**Bioproject accession Col 19 (S)**–**Biosample accession Col 20 (T)**–**16s/18s Sequence ID
MFQPCR	MFQPCR	Col 1–20 (A-T)**–**Design table Col 21 (U)**–**Sample volume (mL) Col 22 (V)**–**Original sample DNA conc. (ng/uL) Col 23 (W)**–**Dilution factor Col 24 (X)**–**Submitted sample DNA conc. (ng/uL) Col 25–39 (Y-AM)**–**Gene abundance (copies/mL) Col 40–54 (AN-BB)**–**Gene abundance (copies/ng DNA) Col 55 (BC)**–**X/End of data record
Water Quality	WQ_nutrients	Col 1–20 (A-T)**–**Design table Col 21 (U)**–**NH_3_ (mg/L) Col 22 (V)**–**BOD (mg/L) Col 23 (W)**–**Chl-a (mg/L) Col 24 (X)**–**NOx (mg/L) Col 25 (Y)**–**Sulphides (mg/L) Col 26 (Z)**–**TSS (mg/L) Col 27 (AA)**–**TKN (mg/L) Col 28 (AB)**–**TN (mg/L) Col 29 (AC)**–**TOC (mg/L) Col 30 (AD)**–**TP (mg/L) Col 31 (AE)**–**Temperature (°C) Col 32 (AF)**–**pH Col 33 (AG)**–**Turbidity (NTU) Col 34 (AH)**–**DO (mg/L) Col 35 (AI)**–**DO (% saturation) Col 36 (AJ)**–**TDS (g/L) Col 37 (AK)**–**Salinity (ppt) Col 38 (AL)**–**X/End of data record

**Table 5:** Dataset record – Ecotox Single-Species Algae.

Data Type	Data File Tab	File Format
Design Table	Mapping	Col 1/2 (A/B)**–**Unique sample ID/name Col 3 (C)**–**Dataset Col 4/5 (D/E)**–**Species/ID Col 6 (F)**–**Date Col 7/8 (G/H)**–**WWTP/ID Col 9/10 (I/J)**–** Latitude/Longitude Col 11/12/13 (K/L/M)**–**Wastewater concentration/code/ID Col 14/15/16 (N/O/P)**–**Water/code/ID Col 17/18 (Q/R)**–**Time point/ID Col 19 (S)**–**Replicate
Cell Counts	FCM_single-species	Col 1–19 (A-S)**–**Design table Col 20 (T)**–**Chlorophyll-a (Fresh) (cells/mL) Col 21 (U)**–**X/End of data record
Water Quality	WQ_nutrients	Col 1–19 (A-S)**–**Design table Col 20 (T)**–**pH Col 21 (U)**–**DO sat% Col 22 (V)**–**NH_3_ (mg/L) Col 23 (W)**–**NOx (mg/L) Col 24 (X)**–**TKN (mg/L) Col 25 (Y)**–**TN (mg/L) Col 26 (Z)**–**TP (mg/L) Col 27 (AA)**–**X/End of data record

**Table 6:** Dataset record – Ecotox Microbial Community.

Data Type	Data File Tab	File Format
Design Table	Mapping	Col 1/2 (A/B)**–**Unique sample ID/name Col 3 (C)**–**Dataset Col 4/5 (D/E)**–**Species/ID Col 6 (F)**–**Date Col 7/8 (G/H)**–**WWTP/ID Col 9/10 (I/J)**–** Latitude/Longitude Col 11/12/13 (K/L/M)**–**Wastewater concentration/code/ID Col 14/15/16 (N/O/P)**–**Water/code/ID Col 17/18 (Q/R)**–**Time point/ID Col 19 (S)**–**Replicate Col 20 (T)**–**Bioproject accession Col 21 (U)**–**16s Sequence ID Col 22 (V)–16s Biosample accession Col 23 (W)–18s Sequence ID Col 24 (X)**–**18s Biosample accession
MFQPCR	MFQPCR	Col 1–19 (A-S)**–**Design table Col 20 (T)**–**Sample volume (mL) Col 21 (U)**–**Original sample DNA conc. (ng/uL) Col 22 (V)**–**Dilution factor Col 23 (W)**–**Submitted sample DNA conc. (ng/uL) Col 24–35 (X-AI)**–**Gene abundance (copies/mL) Col 36–47 (AJ-AU)**–**Gene abundance (copies/ng DNA) Col 48 (AV)**–**X/End of data record
Cell Counts	FCM_microbes	Col 1–19 (A-S)**–**Design table Col 20 (T)**–**Bacteria (Frozen) (cells/mL) Col 21 (U)–Chlorophyll-a (Fresh) (cells/mL) Col 22 (V)–Chlorophyll-a (Frozen) (cells/mL) Col 23 (W)**–**X/End of data record
Water Quality	WQ_nutrients	Col 1–19 (A-S)**–**Design table Col 20 (T)**–**pH Col 21 (U)**–**H_2_S (mg/L) Col 22 (V)**–**DO sat% Col 23 (W)**–**BOD (µmol/L) Col 24 (X)**–**Temperature (°C) Col 25 (Y)**–**NH_3_ (mg/L) Col 26 (Z)**–**NOx (mg/L) Col 27 (AA)**–**TKN (mg/L) Col 28 (AB)**–**TN (mg/L) Col 29 (AC)**–**TP (mg/L) Col 30 (AD)**–**X/End of data record
Water Quality	BOD	Col 1–19 (A-S)**–**Design table Col 20 (T)**–**pH Col 21 (U)**–**H_2_S (mg/L) Col 22 (V)**–**DO sat% Col 23 (W)**–**BOD (µmol/L) Col 24 (X)**–**Temperature (°C) Col 25 (Y)**–**NH_3_ (mg/L) Col 26 (Z)**–**NOx (mg/L) Col 27 (AA)**–**TKN (mg/L) Col 28 (AB)**–**TN (mg/L) Col 29 (AC)**–**TP (mg/L) Col 30 (AD)**–**X/End of data record

## Technical Validation

### Single-species WET tests

The singles-species WET tests fulfilled the standard validity criteria^43^, including less than 20% coefficient of variation; greater than one (1) doublings per day of algal cells in control treatments; and less than 1-unit change in pH over the test duration. Note a reference test using a range of concentrations of copper sulfide (Cu_2_S: 2 µg/L, 4 µg/L, 8 µg/L, 16 µg/L, 32 µg/L and 64 µg/L) was also completed to ensure normal reactivity of the microalgae, and to provide a base for potential comparisons with tests conducted at different facilities. These concentrations were achieved by adding aliquots of a CuSO_4_ stock solution (2 mg/L) to soft-water of moderate hardness (100–120 mg CaCO_3_/L). The reference test using Cu_2_S showed a standard dose response of the algae, thus confirming the validity of the test conditions.

### Flow cytometry

Between samples, fluidics lines were cleared with a wash volume of 500 µL undiluted phosphate-buffered saline (PBS) solution. Additional wash steps using wells containing bleach and then milli-Q water were included between effluent types on each microplate and at the end of each microplate to reduce sample carryover. Positive (stained) and negative (unstained) control samples containing aliquots of river water were also analysed at the end of each microplate for protocol validation.

### DNA extraction

Extracted DNA was quantified using a NanoDrop Spectrophotometer (ThermoFisher Scientific) with concentrations greater than 5–10 ng/ul. To test if the 16S rRNA gene in the different samples could be amplified, PCRs were conducted using the same primer set that was later used for amplicon sequencing (27 f/519r). The PCR reaction mixture (50 μL) contained 10 μL of 5-fold MyTaq Reaction Buffer (Bioline, Alexandria, NSW), 0.4 μM of each primer, 1 U of MyTaq DNA Polymerase (Bioline), and approximately 10 ng of DNA as a template. The following thermal cycling scheme was used: initial denaturation at 95 °C for 2 min, 28 cycles of denaturation at 95 °C for 30 s, annealing at 50 °C for 30 s, followed by extension at 72 °C for 1 min. The final extension was carried out at 72 °C for 2 min. Negative controls were performed by using the reaction mixture without template. Genomic DNA from *Escherichia coli* DH5α was used in the positive control. PCR products were checked with agarose gel electrophoresis. All samples amplified and amplicon size matched the length of the amplified 16 S rRNA gene (approximately 500 bp).

### MFQPCR

The PCR efficiencies of both standards and samples were calculated for each gene using the program LinRegPCR (v2017.1). Only genes with a PCR efficiency of greater than 80% for both standards and samples were further analysed. Further, only those genes with similar PCR efficiencies for the standard and samples (±10%) were used. Afterwards, MFQPCR results were analysed using the Fluidigm Real-Time PCR Analysis program (v4.3.1) using recommended settings (peak sensitivity 7, peak ratio threshold 0.7, quality threshold 0.65). The first analysis step included a manual linear derivative baseline correction. This correction was developed by Fluidigm to correct for baseline drift in real-time PCR data. Ranges of desired melt curve peaks for each primer set were determined manually based on the melt curves of the gBlock standards. Genes with multiple distinguishable melt curve peaks, indicating multiple PCR products due to non-specificity of the primer pair, were excluded from further analyses. Moreover, only samples within the desired melt curve peaks for each primer set were used for further analysis.

Samples were run across two separate 96.96 IFCs; Survey and Ecotox, and quality thresholds were applied separately to each IFC run. A total of 15 primer pairs for genes involved in carbon fixation (rubisco_synecho), nitrogen cycling (napA_3, narG_1, narG, nifD_33, nifH_32 and nirK), sulphur cycling (dsrA, phsA and sir), phosphorous uptake (Acc_ppk1) and pathogen identification (ciaB, ftsZ and stx2) passed the above standard validity tests for the Survey IFC. In addition, the primer pair for the 18S rRNA gene (18S) passed the validity criteria and was included in the analyses as a proxy for the abundance of eukaryotic cells in the samples. However, for genes ciaB and stx2, only single data points (sample-primer combinations) passed the validity tests, thus excluding these two (2) pathogen-related genes from further analyses. For the Ecotox IFC, 12 primer pairs passed the standard validity tests, encompassing the nitrogen cycle (AOA_8, AOB_1, napA_14, napA_3, narG_1, narG, nirS_ef, nrfA_2), sulphur cycling (dsrA, phsA), phosphorous cycle (phoD) and eukaryotes (18s).

## Usage Notes

In this study, bacterial cell densities were quantified in the microbial WET tests from frozen samples several months after the initial flow cytometry was done using fresh samples. Previous studies have reported decreases in bacterial^44^ cell densities between natural and frozen samples. Therefore, these data are recommended for assessment of relative differences between samples since they may reflect a slight underestimation of the bacterial relative to algal cell chlorophyll-a contributions.

While several studies have found that MFQPCR produces copy number estimates that are directly comparable to those produced with traditional qPCR^45,46^, variations in reaction efficiency are common between samples from different sites^47^, and in MFQPCR these differences in efficiency may be substantial^38^. Therefore, whilst copy number tables presented here are suitable for intra-study comparisons and modelling, it is recommended that raw data files are utilised in studies intending to combine inter-study datasets, and uniform efficiency cut-offs be applied prior to further analysis.
